# Supplementary material for: A 12-hospital prospective evaluation of a clinical decision support prognostic algorithm based on logistic regression as a form of machine learning to facilitate decision making for patients with suspected COVID-19
Source: PLoS One. 2022 Jan 5;17(1):e0262193. doi: 10.1371/journal.pone.0262193 (PMC8730444; doi:10.1371/journal.pone.0262193)
Supplement: S1 Table — (DOCX) [file pone.0262193.s001.docx]

**S1 Table.** Factors of interest and factors selected for the final model

| **Factors of interest** | **Factors selected for final model by LASSO** | **Factors included for final model** |
| --- | --- | --- |
| Age | Age | Age |
| Male | Male | Male |
| Race | Race | Race |
| Non-English-speaking | Non-English-speaking | Non-English-speaking |
| Overweight or obese (body mass index [BMI] > 25 | Overweight or obese (body mass index [BMI] > 25 | Overweight or obese (body mass index [BMI] > 25 |
| CCB | CCB | CCB |
| HCTZ | HCTZ | HCTZ |
| BB | BB | BB |
| ACEi | ACEi | ACEi |
| ARB | ARB | ARB |
| Metformin | Metformin | Metformin |
| Warfarin | Warfarin | Warfarin |
| Rivaroxaban | Rivaroxaban | Rivaroxaban |
| Oral Steroids | Oral Steroids | Oral Steroids |
| PPI | PPI | PPI |
| Clopidogrel | Clopidogrel | Clopidogrel |
| Inhaled Steroid | Inhaled Steroid | Inhaled Steroid |
| Aspirin | Aspirin | Aspirin |
| Loop Diuretic | Loop Diuretic | Loop Diuretic |
| Hypertension | New Obese | New Obese |
| T1DM | Hypertension | Hypertension |
| T2DM | T1DM | T1DM |
| CAD | T2DM | T2DM |
| Hx VTE | CAD | CAD |
| Any Heart Fail | Hx VTE | Hx VTE |
| COPD | Any heart fail | Any heart fail |
| Any asthma | COPD | COPD |
| Pacemaker AICD VAD | Any asthma | Any asthma |
| Pulm HTN | Pacemaker AICD VAD | Pacemaker AICD VAD |
| Any CKD | Pulm HTN | Pulm HTN |
| Afib/Aflutter | Any CKD | Any CKD |
| CerebroVascDz | Afib/Aflutter | Afib/Aflutter |
| IBD | CerebroVascDz | CerebroVascDz |
| Rheum arthritis | IBD | IBD |
| Any cancer | Rheum arthritis | Rheum arthritis |
| Sleep apnea | Any cancer | Any cancer |
| HR max24h | Sleep apnea | Sleep apnea |
| RR max24h | HR max 24h | HR max24h |
| Temp max24h | RR max 24h | RR max24h |
| SpO_2_ min 24h | Temp max 24h | Temp max 24h |
| SBP min 24h | SpO_2_ min 24h | SpO_2_ min 24h |
| RDW24 | SBP min 24h | SBP min 24h |
| MCV24 | D dimer max 24h |  |
| ANIONGAP24 | Abs Nphil C24 |  |
| CO_2_24 | RDW24 |  |
| Glucose24 | MCV24 |  |
| PLT24 | PLT24 |  |
| K24 | K24 |  |
| Na24 | CREATININE24 |  |
| D dimer24 | CRP24 |  |
| HGB24 | Abs Nphil Ct24 |  |
| CREATININE24 |  |  |
| CRP24 |  |  |
| Ab Nphil Ct24 |  |  |
| Abs lymph Ct24 |  |  |

**Abbreviations:** CCB: calcium channel blocker; mo: months; BB: betablocker; HCTZ: hydrochlorothiazide; ACEi: angiotensin-converting enzyme inhibitor; ARB: angiotensin receptor blocker; PPI: proton pump inhibitor; T1DM: Type 1 diabetes mellitus; T2DM: Type 2 diabetes mellitus, CAD: coronary artery disease; Hx VTE: history of venous thromboembolism; Heart fail: heart failure; COPD: chronic obstructive lung disease; AICD: automatic implantable cardioverter defibrillator; VAD: ventricular assist device, CKD: chronic kidney disease; Pulm HTN: pulmonary hypertension; Afib: atrial fibrillation; Aflutter: atrial flutter; CerebroVascDz: cerebrovascular disease; IBD: inflammatory bowel disease; rheum: rheumatoid; HR: heart rate; RR: respiratory rate; Temp: temperature; max 24h: maximum within 24 hours; min 24h: minimum within 24 hours; SpO_2_: peripheral arterial oxygen saturation; SBP: systolic blood pressure; RDW: red cell distribution width; MCV: mean corpuscular volume; CO2: carbon dioxide; PLT: platelets; K; potassium; Na: sodium; HGB: hemoglobin; CRP:C-reactive protein; Abs_Nphil_Ct: absolute neutrophil count; Abs_lymph_Ct: absolute lymphocyte count.
