# Supplementary material for: A 12-hospital prospective evaluation of a clinical decision support prognostic algorithm based on logistic regression as a form of machine learning to facilitate decision making for patients with suspected COVID-19
Source: PLoS One. 2022 Jan 5;17(1):e0262193. doi: 10.1371/journal.pone.0262193 (PMC8730444; doi:10.1371/journal.pone.0262193)
Supplement: S2 Table — (DOCX) [file pone.0262193.s002.docx]

**S2 Table.** Categories of Comorbidities and ICD 10 Codes

| **Comorbidity** | **ICD10 Codes** |
| --- | --- |
| **Hypertension** | R03.0, I10, I16.0, I11.0, I15.2, I12.9, I11.0 |
| **T1DM** | E10.21, Z96.41, E10.8, E10.9, E10.10, E10.65, E10.9, E10.641, E10.40, E10.22, E10.69, E10.649, E10.* |
| **T2DM** | E11.* |
| **CAD** | I25.* |
| **Obesity** | E66*, Z68.3* |
| **History of VTE** | I27.82, Z86.711, Z86.718, I82* |
| **Heart failure** | I50* |
| **COPD** | J44*, J41*, J43* |
| **Any asthma** | J45* |
| **Pacemaker AICD/VAD** | Z95.0*, Z95.81* |
| **Pulmonary hypertension** | I27.2*, I27.0*, I27.81, I27.83 |
| **Any CKD** | N18* |
| **Afib/Aflutter** | I48* |
| **Cerebrovascular disease** | G45.*, G46.*, H34.0, I6*.*, I97.81*, I97.82* |
| **IBD** | K50*, K51* |
| **Sleep apnea** | G47.3* |
| **Rheumatoid arthritis** | M06*, M05* |
| **Any ancer** | C**.*, C44.* |
|  |  |

**Abbreviations:** T1DM: Type 1 diabetes mellitus, T2DM: Type 2 diabetes mellitus, CAD: coronary artery disease, VTE: venous thromboembolism, COPD: chronic obstructive lung disease, AICD: automatic implantable cardioverter defibrillator, VAD: ventricular assist device, CKD: chronic kidney disease, Afib: atrial fibrillation, Aflutter: atrial flutter, IBD: inflammatory bowel disease.
