# Supplementary material for: A 12-hospital prospective evaluation of a clinical decision support prognostic algorithm based on logistic regression as a form of machine learning to facilitate decision making for patients with suspected COVID-19
Source: PLoS One. 2022 Jan 5;17(1):e0262193. doi: 10.1371/journal.pone.0262193 (PMC8730444; doi:10.1371/journal.pone.0262193)
Supplement: S3 Table — (DOCX) [file pone.0262193.s003.docx]

**S3 Table.** Distribution of outcomes by score ranges in quintile for the temporal validation data set (n=414)

| **Quintiles of Scores** | **Score Range** | **ICU admission, n(%)** | **Ventilator use, n(%)** | **Death, n(%)** | **N** |
| --- | --- | --- | --- | --- | --- |
| **Lowest 20% scores** | 0 - 0.0104 | 0 (0) | 0 (0) | 0 (0) | 83 |
| **20-40%** | 0.0104-0.025 | 4 (4.8%) | 0 (0) | 0 (0) | 83 |
| **40-60%** | 0.025 - 0.066 | 7 (8.4%) | 0 (0) | 0 (0) | 83 |
| **60-80%** | 0.066 - 0.168 | 13 (15.7%) | 1 (1.2%) | 1 (1.2%) | 83 |
| **Highest 20% of scores** | 0.168-1.0 | 51 (62.2%) | 13 (15.9%) | 6 (7.3%) | 82 |

**Abbreviations:** ICU: Intense Care Unit; Vent: Ventilator.
