# Supplementary material for: A 12-hospital prospective evaluation of a clinical decision support prognostic algorithm based on logistic regression as a form of machine learning to facilitate decision making for patients with suspected COVID-19
Source: PLoS One. 2022 Jan 5;17(1):e0262193. doi: 10.1371/journal.pone.0262193 (PMC8730444; doi:10.1371/journal.pone.0262193)
Supplement: S4 Table — (DOCX) [file pone.0262193.s004.docx]

**S4 Table.** Clinical performance of the logistical model for predicting COVID-19 disease severity* in the temporal validation data set (n=414)

| **Cut points** | **True +** | **False +** | **True -** | **False -** | **Sensitivity** | **Specificity** | **NPV** | **PPV** | **LR +** | **LR -** |
| --- | --- | --- | --- | --- | --- | --- | --- | --- | --- | --- |
| >0.03 | 72 | 157 | 181 | 4 | 94.7% | 53.6% | 0.31 | 0.98 | 2.04 | 0.10 |
| >0.05 | 68 | 121 | 217 | 8 | 89.5% | 64.2% | 0.36 | 0.96 | 2.50 | 0.16 |
| >0.07 | 63 | 94 | 244 | 13 | 82.9% | 72.2% | 0.40 | 0.95 | 2.98 | 0.24 |
| >0.09 | 58 | 74 | 264 | 18 | 76.3% | 78.1% | 0.44 | 0.94 | 3.49 | 0.30 |
| >0.1 | 56 | 68 | 270 | 20 | 73.7% | 79.9% | 0.45 | 0.93 | 3.66 | 0.33 |
| >0.11 | 54 | 60 | 278 | 22 | 71.1% | 82.2% | 0.47 | 0.93 | 4.00 | 0.35 |
| >0.13 | 50 | 48 | 290 | 26 | 65.8% | 85.8% | 0.51 | 0.92 | 4.63 | 0.40 |
| >0.15 | 48 | 40 | 298 | 28 | 63.2% | 88.2% | 0.55 | 0.91 | 5.34 | 0.42 |
| >0.17 | 48 | 32 | 306 | 28 | 63.2% | 90.5% | 0.60 | 0.92 | 6.67 | 0.41 |
| >0.19 | 46 | 27 | 311 | 30 | 60.5% | 92.0% | 0.63 | 0.91 | 7.58 | 0.43 |

* COVID-19 severity is defined as ICU admission, ventilator use, or death.

**Abbreviations:**  True +: True Positive; False +: False Positive; True -: True Negative; False -: False Negative; NPV: Negative Predictive Value; PPV: Positive Predictive Value; LR +: Likelihood Ratio Positive; LR -: Likelihood Ratio Negative.
