# Supplementary material for: A 12-hospital prospective evaluation of a clinical decision support prognostic algorithm based on logistic regression as a form of machine learning to facilitate decision making for patients with suspected COVID-19
Source: PLoS One. 2022 Jan 5;17(1):e0262193. doi: 10.1371/journal.pone.0262193 (PMC8730444; doi:10.1371/journal.pone.0262193)
Supplement: S5 Table — (DOCX) [file pone.0262193.s005.docx]

**S5 Table. Distribution of outcomes by score ranges in quintile for the PUI data set (n=13,271)**

| **Quintiles of Scores** | **Score Range** | **ICU admission n(%)** | **Ventilator use, n(%)** | **Death, n(%)** | **n** |
| --- | --- | --- | --- | --- | --- |
| **Lowest 20% scores** | 0.00062 - 0.0074 | 27 ( 1.0%) | 4 ( 0.3%) | 4 ( 0.2%) | 2,655 |
| **20-40%** | 0.0074 - 0.015 | 70 ( 2.7%) | 14 ( 0.9%) | 13 ( 0.5%) | 2,654 |
| **40-60%** | 0.015 - 0.032 | 128 ( 4.9%) | 31 ( 1.8%) | 21 ( 0.8%) | 2,654 |
| **60-80%** | 0.032 - 0.088 | 368 (14.0%) | 105 ( 5.1%) | 105 ( 4.0%) | 2,654 |
| **Highest 20% of scores** | 0.088 - 0.999 | 835 (31.6%) | 324 (13.8%) | 317 (11.9%) | 2,654 |

**Abbreviations:** ICU: Intense Care Unit; Vent: Ventilator.
