# Supplementary material for: A 12-hospital prospective evaluation of a clinical decision support prognostic algorithm based on logistic regression as a form of machine learning to facilitate decision making for patients with suspected COVID-19
Source: PLoS One. 2022 Jan 5;17(1):e0262193. doi: 10.1371/journal.pone.0262193 (PMC8730444; doi:10.1371/journal.pone.0262193)
Supplement: S6 Table — (DOCX) [file pone.0262193.s006.docx]

**S6 Table. Clinical performance of the logistical model for predicting COVID-19 severity* in the PUI data set (n=13,271)**

| **Cut points** | **True +** | **False +** | **True -** | **False -** | **Sensiti**  **vity** | **Specifi**  **city** | **NPV** | | **PPV** | **LR +** | **LR -** |
| --- | --- | --- | --- | --- | --- | --- | --- | --- | --- | --- | --- |
| >0.03 | 1356 | 3797 | 7847 | 271 | 83.3% | 67.4% | | 0.3 | 1.0 | 2.6 | 0.2 |
| >0.05 | 1162 | 2598 | 9046 | 465 | 71.4% | 77.7% | | 0.3 | 1.0 | 3.2 | 0.4 |
| >0.07 | 1006 | 1950 | 9694 | 621 | 61.8% | 83.3% | | 0.3 | 0.9 | 3.7 | 0.5 |
| >0.09 | 911 | 1513 | 10131 | 716 | 56.0% | 87.0% | | 0.4 | 0.9 | 4.3 | 0.5 |
| >0.1 | 850 | 1390 | 10254 | 777 | 52.2% | 88.1% | | 0.4 | 0.9 | 4.4 | 0.5 |
| >0.11 | 794 | 1247 | 10397 | 833 | 48.8% | 89.3% | | 0.4 | 0.9 | 4.6 | 0.6 |
| >0.13 | 719 | 1031 | 10613 | 908 | 44.2% | 91.1% | | 0.4 | 0.9 | 5.0 | 0.6 |
| >0.15 | 653 | 871 | 10773 | 974 | 40.1% | 92.5% | | 0.4 | 0.9 | 5.4 | 0.6 |
| >0.17 | 590 | 748 | 10896 | 1037 | 36.3% | 93.6% | | 0.4 | 0.9 | 5.6 | 0.7 |
| >0.19 | 540 | 648 | 10996 | 1087 | 33.2% | 94.4% | | 0.5 | 0.9 | 6.0 | 0.7 |

* COVID-19 severity is defined as ICU admission, ventilator use, or death.

**Abbreviations:**  True +: True Positive; False +: False Positive; True -: True Negative; False -: False Negative; NPV: Negative Predictive Value; PPV: Positive Predictive Value; LR +: Likelihood Ratio Positive; LR -: Likelihood Ratio Negative.
