# Supplementary material for: A 12-hospital prospective evaluation of a clinical decision support prognostic algorithm based on logistic regression as a form of machine learning to facilitate decision making for patients with suspected COVID-19
Source: PLoS One. 2022 Jan 5;17(1):e0262193. doi: 10.1371/journal.pone.0262193 (PMC8730444; doi:10.1371/journal.pone.0262193)
Supplement: S1 Fig — (PDF) [file pone.0262193.s007.pdf]

S1 Fig. Implementation of the model for predicting COVID-19 severity in ED

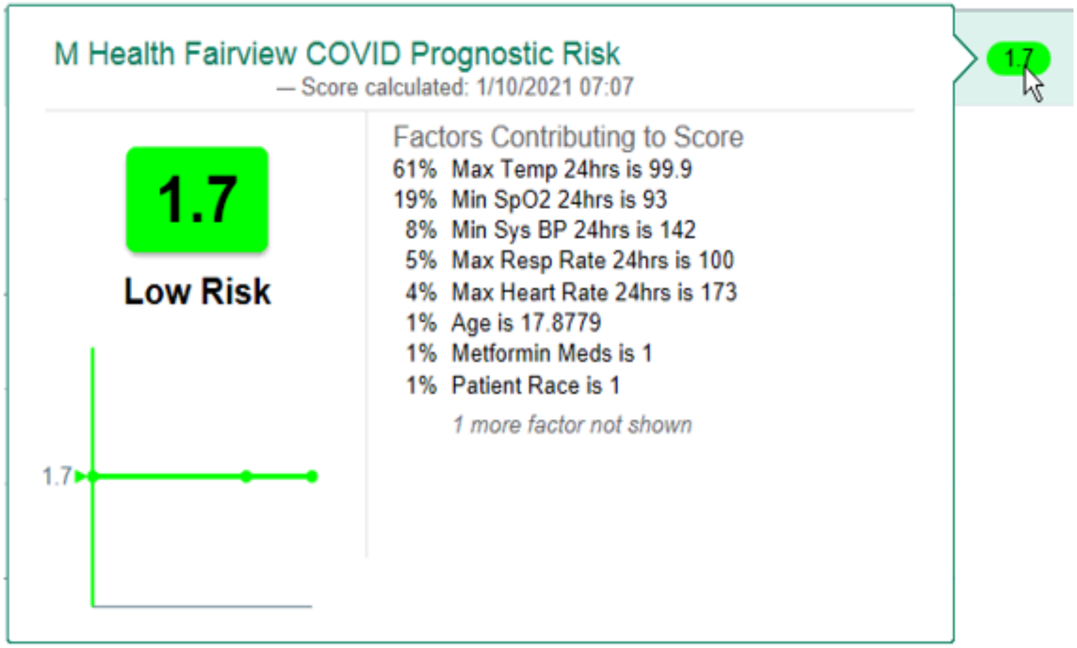

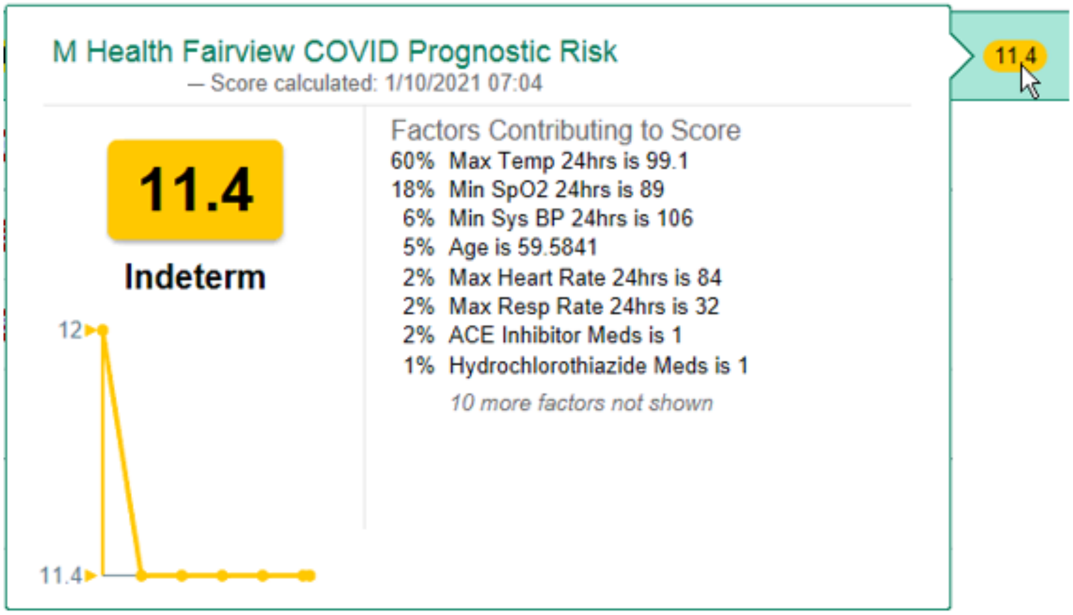

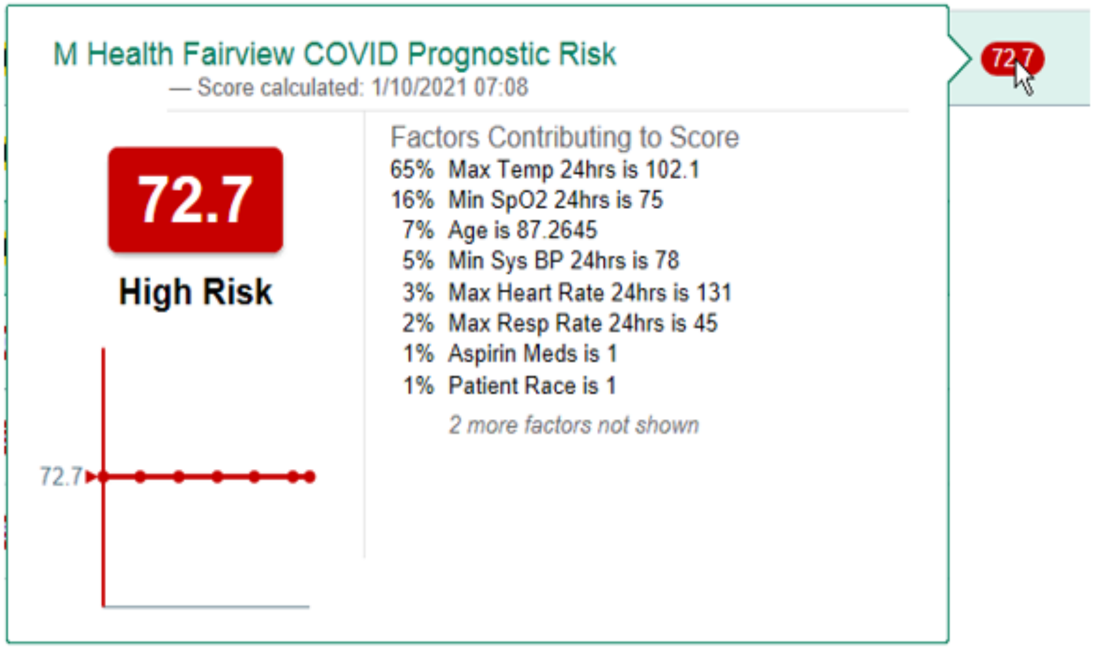

© 2021 Epic Systems Corporation.
