# Supplementary figures and images for: A 12-hospital prospective evaluation of a clinical decision support prognostic algorithm based on logistic regression as a form of machine learning to facilitate decision making for patients with suspected COVID-19
Source: PLoS One. 2022 Jan 5;17(1):e0262193. doi: 10.1371/journal.pone.0262193 (PMC8730444; doi:10.1371/journal.pone.0262193)

**S2 Fig. ROC curve for prospective validation (n=414)**

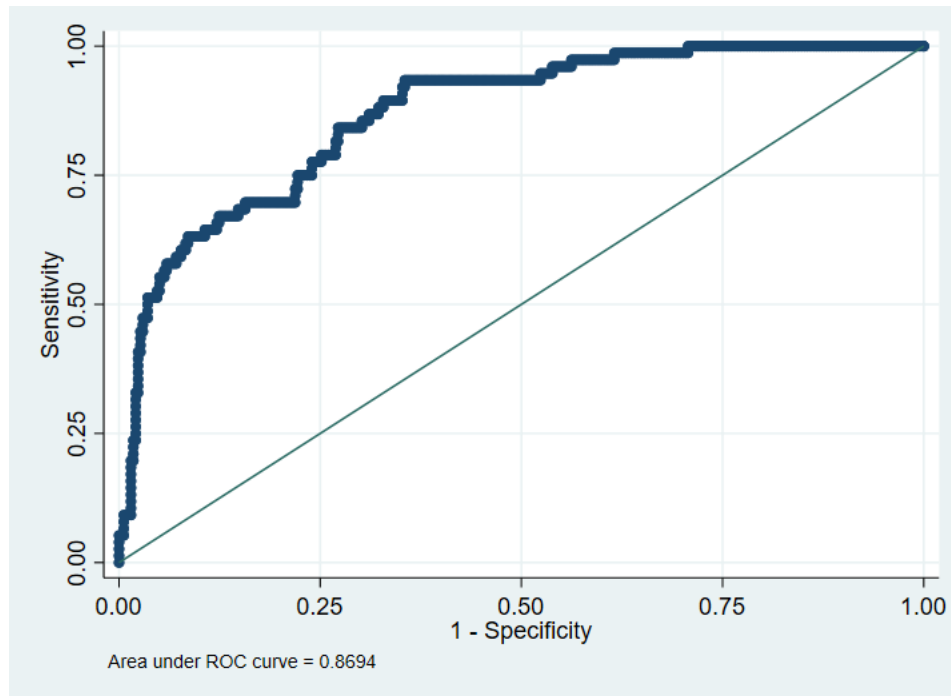

Supplement: S2 Fig — (PDF) [file pone.0262193.s008.pdf]

**S3 Fig. ROC curve for PUI validation (n=13,271)**

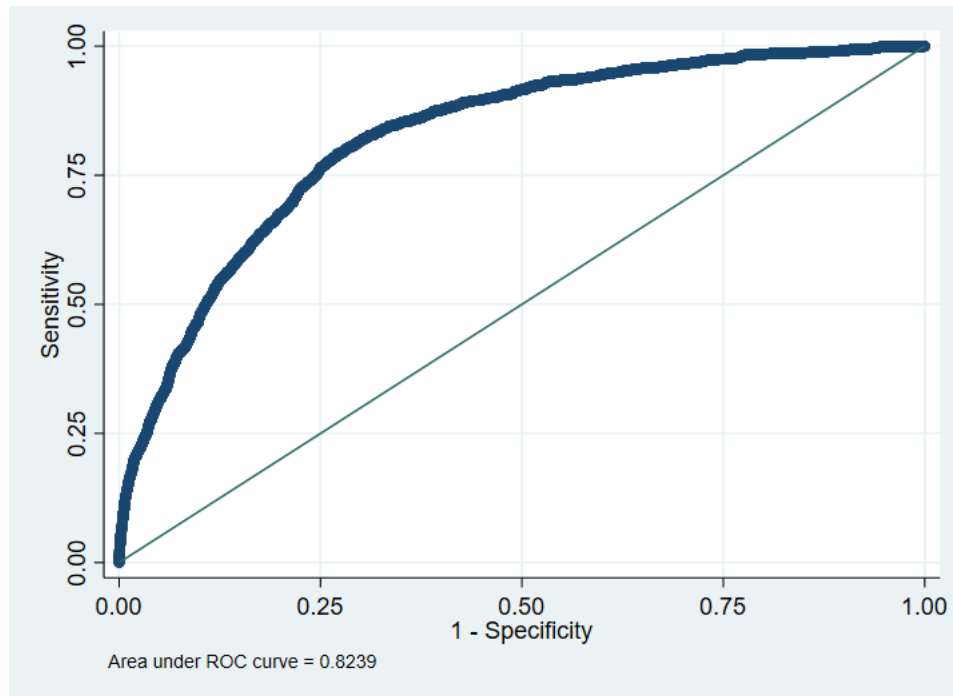

Supplement: S3 Fig — (PDF) [file pone.0262193.s009.pdf]

**S4 Fig. Real-time validation calibration plot**

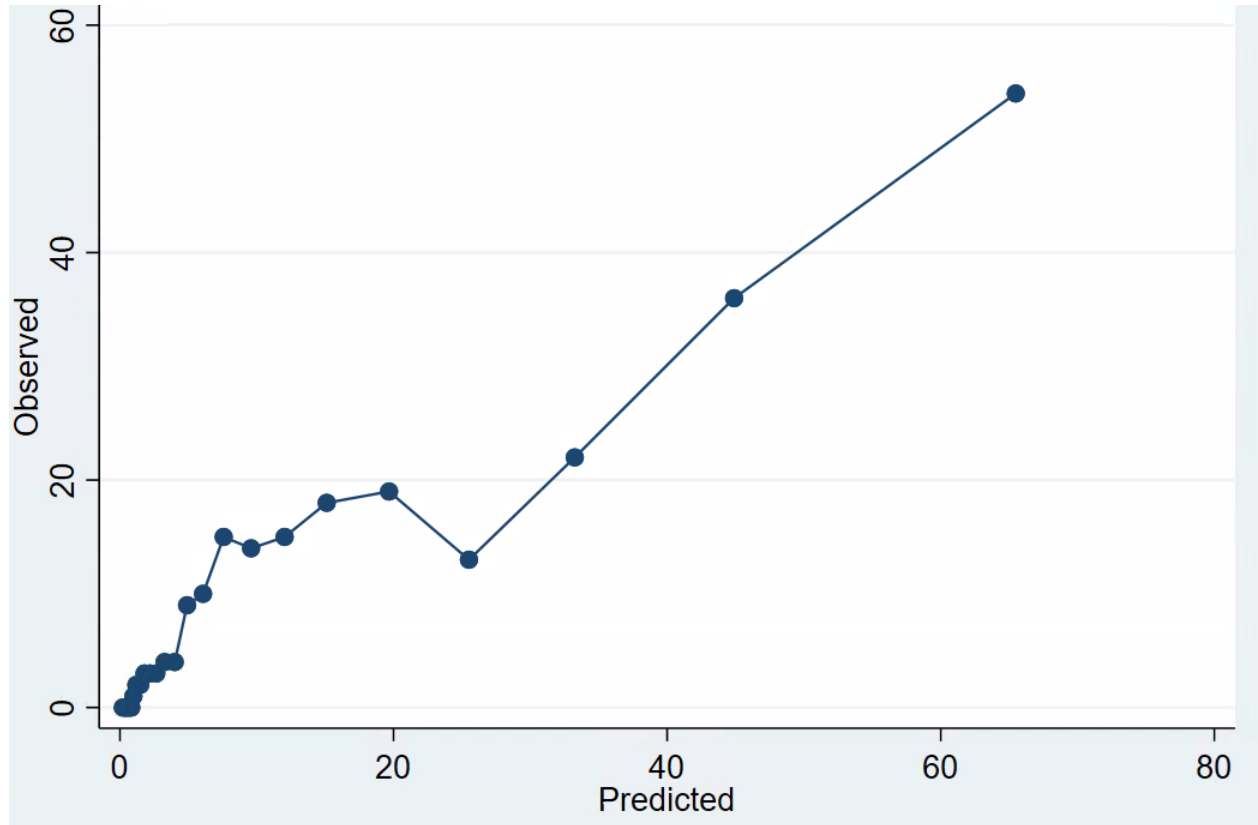

Supplement: S4 Fig — (PDF) [file pone.0262193.s010.pdf]
