# Supplementary material for: A 12-hospital prospective evaluation of a clinical decision support prognostic algorithm based on logistic regression as a form of machine learning to facilitate decision making for patients with suspected COVID-19
Source: PLoS One. 2022 Jan 5;17(1):e0262193. doi: 10.1371/journal.pone.0262193 (PMC8730444; doi:10.1371/journal.pone.0262193)
Supplement: Attachment — Submitted filename: 20211111. Response to PLOS One Reviewers .docx [file pone.0262193.s011.docx]

**Response to PLOS One Editor and Reviewers**

Additional Editor Comments (if provided):

1) Please include all information in Financial Disclosure section as per the PLOS ONE submission guidelines.

Thank you for the kind reminder. We included all information in the Financial Disclosure.

2) All abbreviations in first mention in abstract and introduction must be spelled out.

Thank you for your suggestions. We made the requested changes.

3) The manuscript needs English proof-editing (several typos, misspellings, incomplete sentences, etc)

Thank you for your kind recommendations. We proof-edited the manuscript.

4) The COVID-19 statistics in the introduction need to be updated.

Thank you for your suggestion. We updated the statistics.

**Comments to the Author**

1. Is the manuscript technically sound, and do the data support the conclusions?

The manuscript must describe a technically sound piece of scientific research with data that supports the conclusions. Experiments must have been conducted rigorously, with appropriate controls, replication, and sample sizes. The conclusions must be drawn appropriately based on the data presented.

Reviewer #1: Yes

Reviewer #2: Partly

2. Has the statistical analysis been performed appropriately and rigorously?

Reviewer #1: Yes

Reviewer #2: Yes

3. Have the authors made all data underlying the findings in their manuscript fully available?

The [PLOS Data policy](http://www.plosone.org/static/policies.action#sharing) requires authors to make all data underlying the findings described in their manuscript fully available without restriction, with rare exception (please refer to the Data Availability Statement in the manuscript PDF file). The data should be provided as part of the manuscript or its supporting information, or deposited to a public repository. For example, in addition to summary statistics, the data points behind means, medians and variance measures should be available. If there are restrictions on publicly sharing data—e.g. participant privacy or use of data from a third party—those must be specified.

Reviewer #1: Yes

Reviewer #2: Yes

4. Is the manuscript presented in an intelligible fashion and written in standard English?

PLOS ONE does not copyedit accepted manuscripts, so the language in submitted articles must be clear, correct, and unambiguous. Any typographical or grammatical errors should be corrected at revision, so please note any specific errors here.

Reviewer #1: Yes

Reviewer #2: Yes

5. Review Comments to the Author

Please use the space provided to explain your answers to the questions above. You may also include additional comments for the author, including concerns about dual publication, research ethics, or publication ethics. (Please upload your review as an attachment if it exceeds 20,000 characters)

Reviewer #1: The Authors have designed a model for the severity assessment of COVID-19 patients. The proposed model is simple, efficient and shows good predictive performance. The model has been validated over a very large set of subjects which proves the applicability of the model. The in-depth analysis of the model's performance also gives confidence in the model.

Comments:

1. Test set n=158 statistics not reported in table 1.

Thank you for your suggestion. We introduced the Test set (n=158) characteristics in Table 1.

2. In validation sets B and C, all subjects considered may not be COVID-19 positive (as all patients’ RT-PCR test outcome is unknown). Statistics of how many patients had a positive RT-PCR result must be reported. Further, the severity rate in cohorts B, C are considerably lower than in cohort A.

Thank you for the recommendations. We reported the statistics of how many patients were COVID-19 positive in the PUI and real-time validation sets (we believe that is what you meant by sets B and C), we are happy to further clarify if we misunderstood the reviewer’s comments!

The COVID-19 severity in the training set was higher than the PUI and the real-time data set perhaps because as the pandemic progressed, clinicians better understood how to treat the COVID-19, and the outcomes were better overall as reveled by other studies. As noted below this also likely had to do with our decrease in calibration in the higher risk patient population. While the temporal nature of real-time validation is inevitable, this pandemic is unique in the rapid change in outcomes overtime. This issue is rarely encountered in model prediction; however, despite the change in outcomes overtime, we were pleased that the model continued to perform quite well. This, we view, is another strength of the study. We elaborated on this topic in the Discussion.

3. What criteria were considered for the final list of features to be selected after lasso feature selection?

We included in the final list of features for LASSO only the variables available on presentation to ED. Please see S1 Table.

4. The cut-off points for balanced sensitivity and specificity are around 0.1, which means that the model predictions do not signify real probabilities of severe predictions, limiting the use of the predicted score in the real world. The authors can look into model/probability calibration to fix this.

Thank you for the suggestion. We depicted the calibration plot for the real-time validation data set to evaluate the probabilities in the real world. As seen in the figure, an ideal line for the calibration plot would be a line angled at 45 degrees. Because of imbalance, the higher risk categories do tend to stray slightly from this line. This would indicate that fine tuning is needed at the extremes. This is likely because the “bad outcome” of interest was not as prevalent as it would be needed to create a balanced dataset. This can be improved in future studies along with external validation which would both increase sample size and decrease the calibration effects caused by an imbalanced dataset. We have included the figure in the manuscript along with mentioning this in our limitations in hopes to convey the calibration of our model more adequately.

This was included in the limitations

*“Fourth, as seen in the calibration plot, the model does suffer from at the high-risk end, this is likely due to imbalance of the dataset without a large degree of “bad outcomes”. Future studies will seek to increase sample size and further include external institutions which will aid in further optimization of the model along with addressing the generalizability, respectively.”*

Reviewer #2: This is an interesting manuscript which has obtained a robust COVID-19 prediction model that performs well across gender, race, and ethnicity for three different outcomes. The main problem with this manuscript is that the expected benefit of a CDSS to alleviate burdened and stressed healthcare systems is not demonstrated. This manuscript describes the construction of the model and its predictive capacity, but it has not assessed the impact this model have had on supporting clinical decision making, patient outcomes, or resource use. Authors themselves indicate the need to assess further the benefits of the model presented in this manuscript.

Thank you for your recommendation. We sent a survey to our ED physicians and did not get enough responses in time for submission of this revision. We can include the survey results in further iterations of the manuscript if it is felt that this manuscript would require it for publication. At this time we feel the manuscript does have an adequate amount of information but we are happy to continue pursuing the inclusion of the survey if the reviewers feel the manuscript cannot stand alone without it. To be clear, we feel the survey and its results are vital to better understanding the current results of this study. Our only reservation is if attempting to include the survey would risk the inclusion of too much information for a single manuscript!

6. PLOS authors have the option to publish the peer review history of their article ([what does this mean?](https://journals.plos.org/plosone/s/editorial-and-peer-review-process#loc-peer-review-history)). If published, this will include your full peer review and any attached files.


If you choose “no”, your identity will remain anonymous but your review may still be made public.


**Do you want your identity to be public for this peer review?** For information about this choice, including consent withdrawal, please see our [Privacy Policy](https://www.plos.org/privacy-policy).

Reviewer #1: No

Reviewer #2: No
